# Predicting sample size required for classification performance

**DOI:** 10.1186/1472-6947-12-8

**Published:** 2012-02-15

**Authors:** Rosa L Figueroa, Qing Zeng-Treitler, Sasikiran Kandula, Long H Ngo

**Affiliations:** 1 Dep. Ing. Eléctrica, Facultad de Ingeniería, Universidad de Concepción, Concepción, Chile; 2 Department of Biomedical Informatics, University of Utah, Salt Lake City, Utah, USA; 3 Department of Medicine, Beth Israel Deaconess Medical Center and Harvard Medical School, Boston, MA, USA

## Abstract

**Background:**

Supervised learning methods need annotated data in order to generate efficient models. Annotated data, however, is a relatively scarce resource and can be expensive to obtain. For both passive and active learning methods, there is a need to estimate the size of the annotated sample required to reach a performance target.

**Methods:**

We designed and implemented a method that fits an inverse power law model to points of a given learning curve created using a small annotated training set. Fitting is carried out using nonlinear weighted least squares optimization. The fitted model is then used to predict the classifier's performance and confidence interval for larger sample sizes. For evaluation, the nonlinear weighted curve fitting method was applied to a set of learning curves generated using clinical text and waveform classification tasks with active and passive sampling methods, and predictions were validated using standard goodness of fit measures. As control we used an un-weighted fitting method.

**Results:**

A total of 568 models were fitted and the model predictions were compared with the observed performances. Depending on the data set and sampling method, it took between 80 to 560 annotated samples to achieve mean average and root mean squared error below 0.01. Results also show that our weighted fitting method outperformed the baseline un-weighted method (p < 0.05).

**Conclusions:**

This paper describes a simple and effective sample size prediction algorithm that conducts weighted fitting of learning curves. The algorithm outperformed an un-weighted algorithm described in previous literature. It can help researchers determine annotation sample size for supervised machine learning.

## Background

The availability of biomedical data has increased during the past decades. In order to process such data and extract useful information from it, researchers have been using machine learning techniques. However, to generate predictive models, the supervised learning techniques need an annotated training sample. Literature suggests that the predictive power of the classifiers is largely dependent on the quality and size of the training sample [1-6].

Human annotated data is a scarce resource and its creation expensive both in terms of money and time. For example, un-annotated clinical notes are abundant. To label un-annotated text corpora from the clinical domain, however, requires a group of reviewers with domain expertise and only a tiny fraction of the available clinical notes can be annotated.

The process of creating an annotated sample is initiated by selecting a subset of data; the question is: *what should the size of the training subset be to reach a certain target classification performance? *Or to phrase it differently: *what is the expected classification performance for a given training sample size?*

### Problem formulation

Our interest in sample size prediction stemmed from our experiments with active learning. Active learning is a sampling technique that aims to minimize the size of the training set for classification. The main goal of active learning is to achieve, with a smaller training set, a performance comparable to that of passive learning. In the iterative process, users need to make a decision on when to stop/continue the data labeling and classification process. Although termination criteria is an issue for both passive and active learning, identifying an optimal termination point and training sample size may be more important in active learning. This is because the passive and active learning curves will, given a sufficiently large sample size, eventually converge and thus diminish the advantage of active learning over passive learning. Relatively few papers have been published on the termination criteria for active learning [7-9]. The published criteria are generally based on target accuracy, classifier confidence, uncertainty estimation, and minimum expected error. As such, they do not directly predict a sample size. In addition, depending on the algorithm and classification, active learning algorithms differ in performance and sometimes can perform even worse than passive learning. In our prior work on medical text classification, we have investigated and experimented with several active learning sampling methods and observed the need to predict future classification performance for the purpose of selecting the best sampling algorithm and sample size[10,11]. In this paper we present a new method that predicts the performance at an increased sample size. This method models the observed classifier performance as a function of the training sample size, and uses the fitted curve to forecast the classifier's future behaviour.

### Previous and related work

#### Sample size determination

Our method can be viewed as a type of sample size determination (SSD) method that determines sample size for study design. There are a number of different SSD methods to meet researchers' specific data requirements and goals [12-14]. Determining the sample size required to achieve sufficient statistical power to reject a null hypothesis is a standard approach [13-16]. Cohen defines statistical power as the probability that a test will "yield statistically significant results" i.e. the probability that the null hypothesis will be rejected when the alternative hypothesis is true[17]. These SSD methods have been widely used in bioinformatics and clinical studies [15,18-21]. Some other methods attempt to find the sample size needed to reach a target performance (e.g. a high correlation coefficient) [22-25]. Within this category we find methods that predict the sample size required for a classifier to reach a particular accuracy [2,4,26]. There are two main approaches to predict the sample size required to achieve a specific classifier performance: Dobbin *et al. *describe a "model-based" approach to predict the number of samples needed for classifying microarray data [2]. It determines sample size based on standardized fold change, class prevalence, and number of genes or features on the arrays. Another more generic approach is to fit a classifier's learning curve created using empirical data to inverse power law models. This approach is based on the findings from prior studies where it was shown that the learning classifier learning curves generally follow the inverse power law [27]. Examples of this approach include the algorithms proposed by Mukherjee and others [1,28-30]. Since our proposed method is a variant of this approach, we will describe the prior work on learning curve fitting in more detail.

#### Learning curve fitting

**Figure 1:**
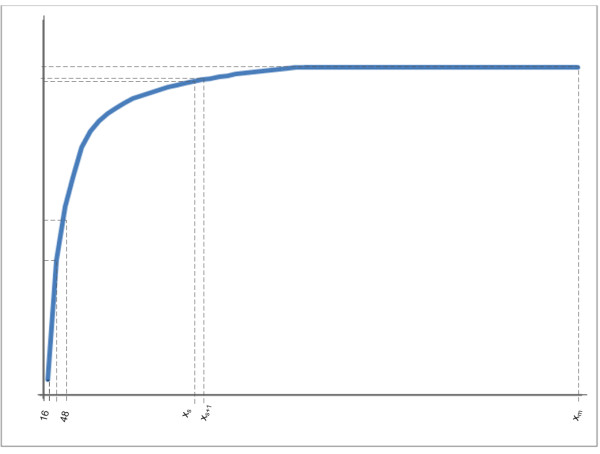
**Generic learning curve**.

A learning curve is a collection of data points (x_j_, y_j_) that in this case describe how the performance of a classifier (y_j_) is related to training sample sizes (x_j_), where j = 1 to m, m being the total number of instances. These learning curves can typically be divided into three sections: In the first section, the classification performance increases rapidly with an increase in the size of the training set; the second section is characterized by a turning point where the increase in performance is less rapid and a final section where the classifier has reached its efficiency threshold, i.e. no (or only marginal) improvement in performance is observed with increasing training set size. Figure 1 is an example of a learning curve.

Mukherjee *et al. *experimented with fitting inverse power laws to empirical learning curves to forecast the performance at larger sample sizes [1]. They have also discussed a permutation test procedure to assess the statistical significance of classification performance for a given dataset size. The method was tested on several relatively small microarray data sets (n = 53 to 280). The differences between the predicted and actual classification errors were found to be in the range of 1%-7%. Boonyanunta *et al. *on the other hand conducted the curve fitting on several much larger datasets (n = 1,000) using a nonlinear model consistent with the inverse power law [28]. The mean absolute errors were very small, generally below 1%. Our proposed method is similar to that discussed in Mukherjee *et al. *with a couple of differences: 1) we conducted weighted curve fitting to favor future predictions; 2) we calculated the confidence interval for the fitted curve rather than fitting two additional curves for the lower and upper quartile data points.

#### Progressive sampling

Another research area related to our work is progressive sampling. Both active learning and progressive sampling start with a very small batch of instances and progressively increase the training data size until a termination criteria is met [31-36]. Active learning algorithms seek to select the most informative cases for training. Several of the learning curves used in this paper were generated using active learning techniques. Progressive sampling, on the other hand, focuses more on minimizing the amount of computation for a given performance target. For instance, Provost *et al. *proposed progressive sampling using a geometric progression-based sampling schedule [31]. They also explored convergence detection methods for progressive sampling and selected a convergence method that used linear regression with local sampling (LRLS). In LRLS, the slope of a linear regression line that has been built with *r *points sampled around the neighborhood of the last sample size is compared to zero. If it is close enough to zero, convergence is detected. The main difference between progressive sampling and SSD of classifiers is that progressive sampling assumes there are an unlimited number of annotated samples and does not predict the sample size required to reach a specific performance target.

## Methods

In this section we describe a new fitting algorithm to predict classifier performance based on a learning curve. This algorithm fits an inverse power law model to a small set of initial points of a learning curve with the purpose of predicting a classifier's performance at larger sample sizes. Evaluation was carried out on 12 learning curves at dozens of sample sizes for model fitting and predictions were validated using standard goodness of fit measures.

### Algorithm description

The algorithm to model and predict a classifier's performance contains three steps:

1) Learning curve creation;

2) Model fitting;

3) Sample size prediction;

#### Learning curve creation

Assuming the target performance measure is classification, a learning curve that characterizes classification accuracy (Y_acc_), as a function of the training set size (X) is created. To obtain the data points (x_j_, y_j_), classifiers are created and tested at increasing training set sizes *x_j_*. With a batch size *k, x _j _*= *k*·*j, j *= 1, 2,...,*m*, i.e. ${\vec{x}}_{j}=\left\{k,2k,3k,...,k\cdot m\right\}$. Classification accuracy points (y_j_), i.e. the proportion of correctly classified samples, can be calculated at each training sample size*x_j _*using an independent test set or through n-fold cross validation.

#### Model fitting and parameter identification

Learning curves can generally be represented using inverse power law functions [1,27,37,38]. Equation (1) describes the classifier's accuracy (Y_acc_) as function of the training sample size × with the parameters a, b, and c representing the minimum achievable error, learning rate and decay rate respectively. The values of the parameters are expected to differ depending on the dataset, sampling method and the classification algorithm. However, values for parameter *c *are expected to be negative within the range [-1,0]; values for *a *are expected to be much smaller than 1. The values of Y_acc _fall between 0 and 1. Y_acc _grows asymptotically to the maximum achievable performance, in this case (1-a).

(1) $$Y_{acc}\left(x\right)=f\left(X;a,b,c\right)=\left(1-a\right)-b\cdot x^{c}$$

Let us define the set Ωas the collection of data points on an empirical learning corresponding *to *($X,Y_{acc_{X}}$). Ω can be partitioned into two sub-sets: Ω*_t _*to fit the model, and Ω*_t _*to validate the fitted model. Please note that in real life applications only Ω*_t _*will be available. For example, at sample size x_s _Ω*_t _*= {(*x*j, *y_j_*)| *x_j _*≤ *x_s_*} and Ω*_v _*= {(*x*j, *y_j_*)| *x_j _*>*x_s_*}.

UsingΩ*_t_*, we applied nonlinear weighted least squares optimization together with the nl2sol routine from Port Library[39] to fit the mathematical model from Eq(1) and find the parameter vector $\vec{\beta }$ = {a, b, c}.

We also assigned weights to the data points inΩ*_t_*. As described earlier, data points on the learning curve associates with sample sizes; we postulated that the classifier performance at a larger training sample size is more indicative of the classifier's future performance. To account for this, a data point (*x_j_, y_j_*)∈Ω*_t _*is assigned the normalized weight *j/m *where *m *is the cardinality of Ω.

#### Performance prediction

In this step, the mathematical model (Eq.(1)) together with the estimated parameters {a, b, c} are applied to unseen sample sizes and the resulting prediction is compared with the data points in Ω*_v_*. In other words, the fitted curve is used to extrapolate the classifier's performance at larger sample sizes. Additionally, the 95% confidence interval of the estimated accuracy${ŷ}_{s}$ is also calculated by using Hessian matrix and the second-order derivatives on the function describing the curve. See appendix1 (additional file 1) for more details on the implementation of the methods.

### Evaluation

#### Datasets

We evaluated our algorithm using three sets of data. In the first two sets (D1 and D2), observations are smoking-related sentences from a set of patient discharge summaries from the Partners Health Care's research patient data repository (RPDR). Each observation was manually annotated with smoking status. D1 contains 7,016 sentences and 350 word features to distinguish between *smokers (5,333 sentences) and non smokers (1,683 sentences)*. D2 contains 8,449 sentences, 350 word features to discriminate between *past smokers (5,109 sentences) and current smokers (3,340 sentences)*.

The third data set (D3) is the *waveform-5000 dataset *from the UCI machine learning repository [40] which contains 5,000 instances, 21 features and three classes of waves (1657 instances of w1, 1647 of w2, and 1696 of w3). The classification goal is to perform binary classification to discriminate the first class of waves from the other two.

Each dataset was randomly split into a training set and a testing set. Test sets for D1 and D2 contained 1,000 instances each while 2,500 instances were set apart as test set in D3. On the three datasets, we used 4 different sampling methods - three active learning algorithms and a random selection (passive) - together with a support vector machine classifier with linear kernel from WEKA [41] (complexity constant was set to 1, epsilon set to 1,0 E-12, tolerance parameter 1,0E-3, and normalization/standardization options were turned off) to generate a total of 12 actual learning curves for Y_acc_. The active learning methods used are:

• Distance (DIST), a simple margin method which samples training instances based on their proximity to a support vector machine (SVM) hyperplane;

• Diversity (DIV) which selects instances based on their diversity/dissimilarity from instances in the training set. Diversity is measured as the simple cosine distance between the candidate instances and the already selected set of instances in order to reduce information redundancy; and

• Combined method (CMB) which is a combination of both DIST and DIV methods.

The initial sample size is set to 16 with an increment size of 16 as well, i.e. k = 16. Detailed information about the three algorithms can be found in appendix 2 (see additional file 2) and in literature [10,35,42].

Each experiment was repeated 100 times and *Y*_*acc *_averaged at each batch size over the 100 runs to obtain data points(*x_j_, y_j_*) of the learning curve.

#### Goodness of fit measures

Two goodness of fit measurements, mean absolute error (MAE) (Eq.(2)) and root mean squared error (RMSE) (Eq.(3)), were used to evaluate the fitted function onΩ*_v_*. MAE is the average absolute value of the difference between the observed accuracy (*y_j_*) and the predicted accuracy (${\overset{⌢}{y}}_{j}$). RMSE is the average of the square root values of the difference between the observed accuracy (*y_j_*) and the predicted accuracy (${\overset{⌢}{y}}_{j}$). RMSE and MAE values of close to zero indicate a better fit. Using ||Ω*_v_*||to represent the cardinality of Ω_v_, MAE and RMSE are computed as follows:

(2) $$MAE=\frac{1}{|{\text{Ω}}_{v}|}\overset{m}{\underset{\left(x_{j},y_{j}\right)\in {\text{Ω}}_{v}}{\sum }}|y_{j}-{\overset{\wedge }{y}}_{j}|\;,\;\forall \;\left(x_{j,}y_{j}\right)\;\in \;{\text{Ω}}_{v}$$

(3) $$RMSE=\sqrt{\frac{\overset{m}{\underset{\left(x_{j},y_{j}\right)\in {\text{Ω}}_{v}}{\sum }}{\left(y_{j}-\overset{\wedge }{y_{j}}\right)}^{2}}{|{\text{Ω}}_{v}|}},\forall \;\left(x_{j},y_{j}\right)\;\in \;{\text{Ω}}_{v}$$

On each curve, we started the curve fitting and prediction experiment at |Ω*_t_*| = 5, i.e. at the sample size of 80 instances. In the subsequent experiments, the |Ω*_t_*| was increased by 1 until it reached 62 points, i.e. at the sample size of 992 instances.

To evaluate our method, we used as baseline the non-weighted least squares optimization algorithm described by Mukherjee *et al *[1]. Paired t-test was used to compare the RMSE and MAE between both methods for all experiments. The alternative hypothesis is that the means of the RMSE and MAE of the baseline method is greater than those of our weighted fitting method.

## Results

Using the 3 datasets and 4 sampling methods, 12 actual learning curves are generated. We fitted the inverse power law model to each of the curves, using an increasing number of data points (m = 80-992 in D1 and D2, m = 80-480 in D3). A total of 568 experiments were conducted. In each experiment, the predicted performance was compared to the actual observed performance.

**Figure 2:**
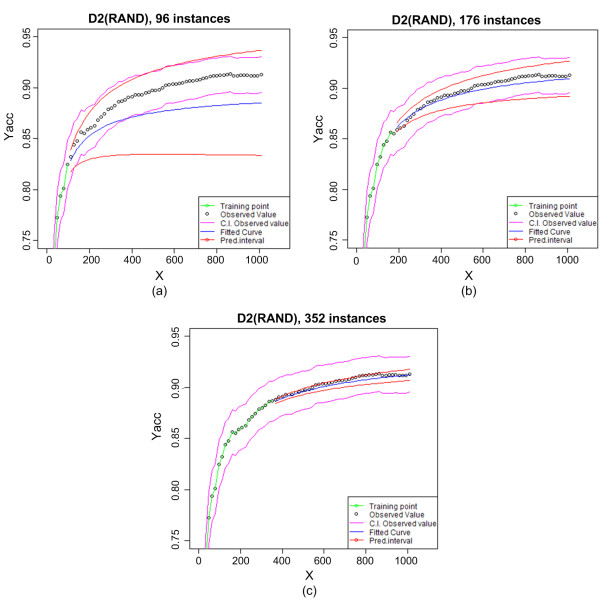
**Progression of online curve fitting for learning curve of the dataset D2-RAND**.

Figure 2 shows the curve fitting and prediction results for the random sampling learning curve using D2 data at different sample sizes. In Figure 2a the curve was fitted using 6 data points; the predicted curve (blue) deviates slightly from the actual data points (black), though the actual data points do fall in the relatively large confidence interval (red). As expected, the deviation and confidence interval are both larger as we project further into the larger sample sizes. In 2b, with 11 data points for fitting, the predicted curve closely resembles the observed data and the confidence interval is much narrower. In 2c with 22 data points, the predicted curve is even closer to the actual observations with a very narrow confidence interval.

**Figure 3:**
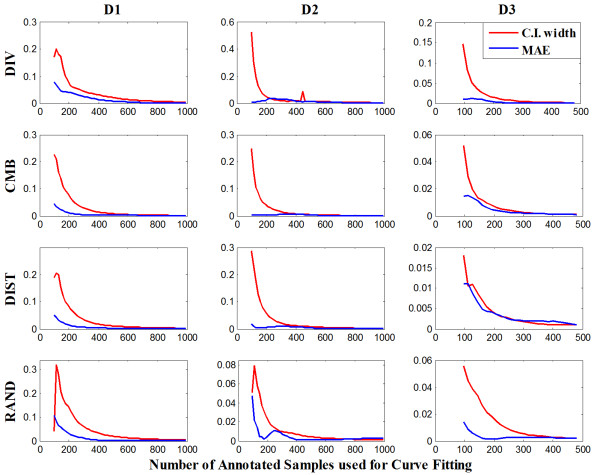
**Progression of confidence interval width and MAE for predicted values**.

Figure 3 illustrates the width of the confidence interval and MAE at various sample sizes. When the model is fitted with a small number of annotated samples, we can observe that the confidence interval width and MAE in most of the cases have larger values. As the sample size increases and the prediction accuracy improves, both confidence interval width and MAE values become smaller within a couple of exceptions. At large sample sizes, confidence intervals are very narrow and residual values very small. Both Figures 2 and 3 suggest that the confidence interval width relates to MAE and prediction accuracy.

**Figure 4:**
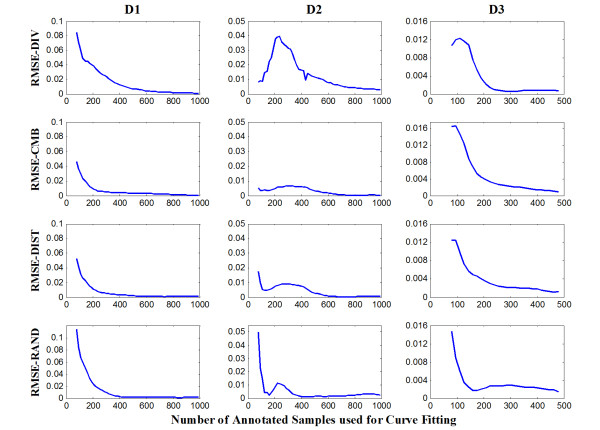
**RMSE for predicted values on the three datasets**.

Similarly, Figure 4 shows RMSE for the predicted values on the 12 learning curves with gradually increasing sample sizes used for curve fitting. Regarding fitting samples sizes, we can observe a rapid decrease in RMSE and MAE from 80 to 200 instances. From 200 to the end of the curves, values stay relatively constant and close to zero with a few exceptions. The smallest MAE and RMSE were obtained from the D3 dataset on all the learning curves, followed by the learning curves on the D2 dataset. For all datasets RMSE and MAE have similar values with RMSE sometimes being slightly larger.

**Figure 5:**
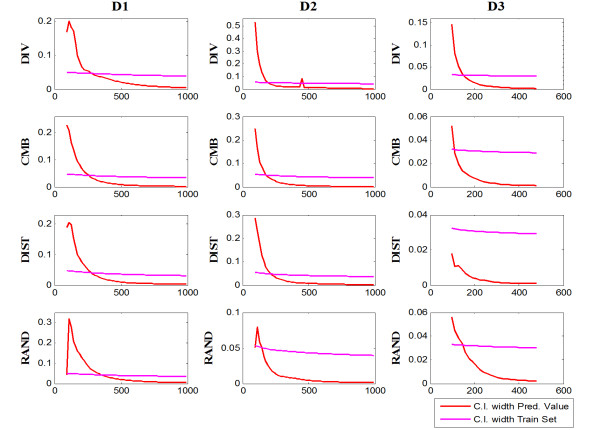
**Progression of confidence interval widths for the observed values (training set) and the predicted values**.

On Figure 2 and 5, it can be observed that the width of the observed confidence intervals changes only slightly along the learning curves, showing that performance variance among experiments are not strongly impacted by the sample size. On the other hand, the predicted confidence interval narrows dramatically as more samples are used and the prediction becomes more accurate.

We also compared our algorithm with the un-weighted algorithm. Table 1 shows average values of RMSE for the baseline un-weighted and our weighted method; min and max values are also provided. In all cases, our weighted fitting method had lower RMSE than baseline method with the exception of one tie. We pooled the RMSE values and conducted a paired t-test. The difference between the weighted fitting method and the baseline method is statistically significant (p < 0.05). We conducted a similar analysis comparing the MAE between the two methods and obtained similar results.

**Table 1:** Average RMSE (%) for baseline and weighted fitting method.

	Average RMSE (%)		
	**Weighted** **[min-max]**	**Baseline** **[min-max]**	**P**

**D1-DIV**	1.52 [0.04 - 8.44]	2.57 [0.82 - 8.70]	2.7E-44

**D1-CMB**	0.60 [0.06 - 4.61]	1.15 [0.44 - 4.94]	2.7E-32

**D1-DIS**	0.61 [0.09 - 5.25]	1.16 [0.22 - 5.50]	1.9E-22

**D1-RND**	1.15 [0.10 - 11.37]	2.01 [0.38 - 11.29]	8.2E-19

**D2-DIV**	1.33 [0.28-3.95]	1.63 [0.73-3.53]	4.6E-09

**D2-CMB**	0.29 [0.01-0.67]	0.38 [0.19-0.76]	3.3E-04

**D2-DIST**	0.39 [0.04-1.74]	0.50 [0.22-2.11]	2.7E-03

**D2-RND**	0.46 [0.13 - 4.99]	0.56 [0.16 - 4.44]	6.1E-04

**D3-DIV**	0.34 [0.05 - 1.22]	0.43 [0.04 - 0.93]	4.6E-02

**D3-CMB**	0.47 [0.09 - 1.66]	0.65 [0.21 - 1.60]	6.0E-09

**D3-DIS**	0.38 [0.10 - 1.24]	0.49 [0.20 - 1.21]	5.1E-10

**D3-RND**	0.32 [0.15 - 1.48]	0.32 [0.11 - 1.75]	6.3E-01

Paired Student's t-test conducted on the values of RMSE found the weighted fitting method statistically better than the baseline method (p < 0.05).

## Discussion

In this paper we described a relatively simple method to predict a classifier's performance for a given sample size, through the creation and modelling of a learning curve. As prior research suggests, the learning curves of machine classifiers generally follow the inverse-power law [1,27]. Given the purpose of predicting future performance, our method assigned higher weights to data points associated with larger sample size. In evaluation, the weighted methods resulted in more accurate prediction (p < 0.05) than the un-weighted method described by Mukherjee et al.

The evaluation experiments were conducted on free text and waveform data, using passive and active learning algorithms. Prior studies typically used a single type of data (e.g. microarray or text) and a single type of sampling algorithm (i.e. random sampling). By using a variety of data and sampling methods, we were able to test our method on a diverse collection of learning curves and assess its generalizability. For the majority of curves, the RMSE fell below 0.01, within a relative small sample size of 200 used for curve fitting. We observed minimal differences between values of RMSE and MAE which indicates a low variance of the errors.

Our method also provides the confidence intervals of the predicted curves. As shown in Figure 2, the width of the confidence interval negatively correlates with the prediction accuracy. When the predicted value deviates more from the actual observation, the confidence interval tends to be wider. As such, the confidence interval provides an additional measure to help users make the decision in selecting a sample size for additional annotation and classification. In our study, confidence intervals were calculated using a variance-covariance matrix on the fitted parameters. Prior studies have stated that the variance is not an unbiased estimator when a model is tested on new data [1]. Hence, our confidence intervals may sometimes be optimistic.

A major limitation of the methods is that an initial set of annotated data is needed. This is a shortcoming shared by other SSD methods for machine classifiers. On the other hand, depending on what confidence interval is deemed acceptable, the initial annotated sample can be of moderate size (e.g. *n *= 100~200).

The initial set of annotated data is used to create a learning curve. The curve contains

*j *data points with a starting sample size of *m_0 _*and a step size of *k*. The total sample size m = m_0 _+ (j-1)*k. The values of *m_0 _*and *k *are determined by users. When *m_0 _*and *k *are assigned the same value, m = j*k. In active learning, a typical experiment may assign *m_0 _*as 16 or 32 and *k *as 16 or 32. For very small data sets, one may consider use *m_0 _*= 4 and *k *= 4. Empirically, we found that *j *needed to be greater than or equal to 5 for the curve fitting to be effective.

In many studies, as well as ours, the learning curves appear to be smooth because each data point on the curve is assigned the average value from multiple experiments (e.g. 10-fold cross validation repeated 100 times). With fewer experiments (e.g. 1 round of training and testing per data point), the curve will not be as smooth. We expect the model fitting to be more accurate and the confidence interval to be narrower on smoother curves, though the fitting process remains the same for the less smooth curves.

Although the curve fitting can be done in real time, the time to create the learning curve depends on the classification task, batch size, feature number, processing time of the machine among others. The longest experiment we performed to create a learning curve using active learning as sample selection method run on a single core laptop for several days, though most experiments needed only a few hours.

For future work, we intend to integrate the function to predict sample size into our NLP software. The purpose is to guide users in text mining and annotation tasks. In clinical NLP research, annotation is usually expensive and the sample size decision is often made based on budget rather than expected performance. It is common for researchers to select an initial number of samples in an ad hoc fashion to annotate data and train a model. They then increase the number of annotations if the target performance could not be reached, based on the vague but generally correct belief that performance will improve with a larger sample size. The amount of improvement though cannot be known without the modelling effort we describe in this paper. Predicting the classification performance for a particular sample size would allow users to evaluate the cost effectiveness of additional annotations in study design. Specifically, we plan for it to be incorporated as part of an active learning and/or interactive learning process.

## Conclusions

This paper describes a simple sample size prediction algorithm that conducts weighted fitting of learning curves. When tested on free text and waveform classification with active and passive sampling methods, the algorithm outperformed the un-weighted algorithm described in previous literature in terms of goodness of fit measures. This algorithm can help users make an informed decision in sample size selection for machine learning tasks, especially when annotated data are expensive to obtain.

## Competing interests

The authors declare that they have no competing interests.

## Authors' contributions

QZ and RLF conceived the study. SK and RLF designed and implemented experiments. SK and RLF analyzed data and performed statistical analysis. QZ and LN participated in study design and supervised experiments and data analysis. RLF drafted the manuscript. Both SK and QZ had full access to all of the data and made critical revisions to the manuscript. All authors read and approved the final manuscript.

## Pre-publication history

The pre-publication history for this paper can be accessed here:

http://www.biomedcentral.com/1472-6947/12/8/prepub

## Supplementary Material

Additional file 1 **Appendix1 is a PDF file with the main lines of R code that implements curve fitting using inverse power models**. Click here for file

Additional file 2 **Appendix 2 is a PDF file that contains more details about the active learning methods used to generate the learning curves**. Click here for file
