# Supplementary material for: Predicting sample size required for classification performance
Source: BMC Med Inform Decis Mak. 2012 Feb 15;12:8. doi: 10.1186/1472-6947-12-8 (PMC3307431; doi:10.1186/1472-6947-12-8)
Supplement: Additional file 1 — Appendix1 is a PDF file with the main lines of R code that implements curve fitting using inverse power models. [file 1472-6947-12-8-S1.PDF]

```
#####################################################################
#####################################################################
#need to set the following parameters
#functions to calculate rmse and mae should be defined by the user
#offset = offset to start fitting, it's always set to zero
#i = number of points to include in training data
#W = weights
#N = Total number of points
#startParams = start parameters to nls function currently set to (a=0,b=1,c=-0.5)


FitModel<- function(offset, X, Y, W, i, N,startParams)
{
      #Data considering only points between offset and i
        x<-X[offset:i];
        y<-Y[offset:i];
        w<-W[offset:i];

      gradientF<-deriv3(~(1-a)-(b*(x^c)), c("a","b","c"), function(a,b,c,x) NULL);

      # fitting the model using nls

      m<-nls(y~gradientF(a,b,c,x), start = startParams, weights=w,
                control = list(maxiter=1000, warnOnly = TRUE),
                algorithm = "port", upper = list(a=10, b = 10, c = -0.1),
                lower = list(a = 0, b = 0, c=-10), data = data.frame(y=y, x=x))


      #predict Y for sample sizes not used to fit the curve
        #if all data was used to fit model, testing data = training data
        #else, testing data  = (total data - training data)


      if (i==N){
           testX<-X[(offset:i)];
           testY<-Y[(offset:i)];
           testW <- W[offset:i];
        }

        else{
          testX<-X[((i+1):N)];           #Get remaining X
          testY<-Y[((i+1):N)];
          testW<-W[((i+1):N)];
        }

       #predictions on unseen data

        prediction<-predict(m, list(x=testX));

        #confidence intervals

        se.fit <- sqrt(apply(attr(predict(m, list(x=testX)),"gradient"),1,
                        function(x) sum(vcov(m)*outer(x,x))));

        prediction.ci <- prediction + outer(se.fit,qnorm(c(.5, .025,.975)));

        predictY<-prediction.ci[,1];
        predictY.lw<-prediction.ci[,2];
        predictY.up<-prediction.ci[,3];
```

```
        #Calculate residuals
      if (i==N)
                 res<-rep(0,length(X))
       else
                 res<-rep(0,length(X-i))
        res<-(predictY-testY);
        #Calculate Root Mean square error
        rmseValue<-rmse(testY, predictY);
        #Calculate Absolute error
        maeValue<-abse(testY, predictY);

}
#End function
```
