# Supplementary material for: Predicting sample size required for classification performance
Source: BMC Med Inform Decis Mak. 2012 Feb 15;12:8. doi: 10.1186/1472-6947-12-8 (PMC3307431; doi:10.1186/1472-6947-12-8)
Supplement: Additional file 2 — Appendix 2 is a PDF file that contains more details about the active learning methods used to generate the learning curves. [file 1472-6947-12-8-S2.PDF]

Additional file 2 – Appendix 2

Active learning algorithms are differentiated by the decision functions used to select instances for labeling. The algorithms in our study used a common decision function

$$\underset{x_i \in U}{\arg\min}\left(\lambda * \left| f(x_i) \right| + (1-\lambda) * \left(\max_{x_j \in S} \left(\text{cos - sim}(x_{i,} x_{j}) \right) \right),\ \lambda \in [0,1] \right)$$

where U represents the unlabeled pool, S the selected samples and λ is the tuning parameter. We implemented 3 variations of the decision function discussed above. With 1) DIST (λ=1), only the first part of the decision function is considered; *f (xi)* represents the distance between a candidate instance x_i in the unlabelled pool U and the classification boundary given by an SVM hyperplane. The selected instances are the ones closest to the classification hyperplane, i.e. the most ambiguous cases. With 2) *DIV(* λ=0), only the second part of the function is considered; *cos-sim* represents the maximum cosine distance x_i and the already selected set of instances, i.e. we select instances that maximize the diversity of the selected sample in order to reduce redundancy among the selected examples. Finally, with 3) *CMB (*λ=0.5) we consider both distance and diversity, i.e. we select instances that are close to the hyperplane and increase diversity.
